# Isolation and mutation trend analysis of influenza A virus subtype H9N2 in Egypt

**DOI:** 10.1186/1743-422X-9-173

**Published:** 2012-08-27

**Authors:** Ahmed S Abdel-Moneim, Manal A Afifi, Magdy F El-Kady

**Affiliations:** 1 Department of Virology, Faculty of Veterinary Medicine, Beni-Suef University, Beni-Suef, 62511, Egypt; 2 Division of Virology, Department of Microbiology, College of Medicine, Taif University, Al-Taif, Saudi Arabia; 3 Department of Poultry Diseases, Faculty of Veterinary Medicine, Cairo University, Giza, Egypt; 4 Department of Poultry Diseases, Faculty of Veterinary Medicine, Beni-Suef University, Beni-Suef, 62511, Egypt

## Abstract

**Background:**

Avian influenza virus H9N2 is a panzootic pathogen that affects poultry causing mild to moderate respiratory distress but has been associated with high morbidity and considerable mortality. Interspecies transmission of H9N2 from avian species to mammalian hosts does occur. The virus possesses human virus-like receptor specificity and it can infect humans producing flu-like illness.

**Methods:**

Recently, mild influenza like symptoms were detected in H5N1 vaccinated flocks. Influenza A subtype H9N2 was isolated from the infected flock. The virus evolution was investigated by sequencing the viral genes to screen the possible virus recombination. The viral amino acid sequences from the isolated H9N2 strains were compared to other related sequences from the flu data base that were used to assess the robustness of the mutation trend. Changes in the species-associated amino acid residues or those that enabled virulence to mammals were allocated.

**Results:**

Phylogenetic analyses of haemagglutinin and neuraminidase genes showed that the recently isolated Egyptian strain belonged to the H9N2 sub-lineage that prevails in Israel. The six internal segments of the isolated virus were found to be derived from the same sub-lineage with no new evidence of reassortment. The results demonstrated conserved genetic and biological constitution of H9N2 viruses in the Middle East. The recently isolated H9N2 virus from chicken in Egypt possessed amino acids that could enable the virus to replicate in mammals and caused severe disease in domestic chickens.

**Conclusion:**

The study highlights the importance of continuous monitoring of the mutations evolved in avian influenza viruses and its impact on virulence to avian species in addition to its importance in the emergence of new strains with the capacity to be a pandemic candidate.

## Background

Influenza A viruses are represented in dozens of antigenic subtypes; 17 haemagglutinin (HA) and 10 neuraminidase (NA) subtypes [1]. After the transfer of the influenza viruses from the aquatic birds to a new avian or mammalian host, the viruses evolve rapidly and cause mild or occasionally severe respiratory disease [2]. H9N2 viruses have been panzootic in the Middle East and Asia over the last decade and have been recorded in different types of poultry worldwide [3]. H9N2 viruses are low pathogenic avian influenza (LPAI); however, they induce significant disease in poultry and are occasionally accompanied by considerable mortality [4]. They are represented by two main lineages defined by the prototype viruses A/Dk/HK/Y280/97 and A/Qa/HK/G1/97 that circulated in the terrestrial poultry in Asia [5]. A/Qa/HK/G1/97-like viruses have been involved in the generation of the highly pathogenic H5N1 virus in 1997 [6]. H9N2 influenza viruses isolated from 1998 to 2010 in Central Asia and the Middle East are clustered in four distinct groups (A-D). Groups A and B have circulated extensively in Central Asia and the Middle East, but groups C and D are restricted to certain countries [7].

H9N2 influenza virus is endemic in poultry all over Eurasia and has transmitted to mammals [8]. The virus possesses human virus-like receptor specificity, and it can infect humans producing flu-like illness [9]. Direct human infections with avian H9N2 confirm that interspecies transmission of H9N2 from avian species to mammalian hosts occurs, and it is not uncommon [10]. The disease in human is usually subclinical [11], and therefore the virus has the opportunity to circulate and reassort with other influenza viruses.

Inter-subtype reassortments have been detected between the co-circulating H9N2 and the highly pathogenic H5N1 or H7N3 viruses [5,12]. These finding together with the endemic nature of the H5N1 virus infection in Egypt [13] make the genetic characterization of other avian viruses circulating in Egypt important for screening the probable reassortment and/or the dynamic evolution of new strains with increased virulence to mammals. The circulation of avian viruses among populations in different parts of the world increases the genetic diversity of influenza viruses [14]. Reassortment between the human pandemic strains and an avian virus of a different subtype is not uncommon [2]. The consequences of the interspecies transmission can be devastating with all catastrophic consequences.

Here, we describe the isolation of the H9N2 virus from a broiler- breeder farm in the northern part of Egypt. The mutation trend and the genetic evolution of the isolated strain were also studied in comparison to other similar influenza virus serotype; H9N2 in the Middle East, the Egyptian H5N1 strains as well the H1N1 recent pandemic strains.

## Methods

### Ethics statement

The use of animal subjects in this study was approved by the Ethical Committee of Beni-Suef University.

### Clinical data

On 5^th^ May 2011, a six-week-old chicken (broiler-breeder) flock in the Alexandria Governorate, Egypt suffered from respiratory distress and facial oedema with low mortality rate (50/10,000). The general health signs of the flock deteriorated rapidly within 2-3 days to chronic respiratory disease (CRD). Morbidity reached 70% while mortality reached 15% with a weak response to antibiotics. The clinical and post-mortem findings revealed severe tracheitis, cyanosis of the head and patches of congestion in the shanks particularly under the hock joint as well as pericarditis, perihepatitis, peritonitis and air-saculitis. The overall body weights and feed conversion rate were significantly affected.

### Sampling and virus isolation

Ten paired tracheal and cloacal swab samples were collected in a sterile phosphate buffer saline during the general surveillance of influenza viruses in Egypt. Swabs were routinely processed individually. Infected materials were pooled, centrifuged at 500 × g for 10 min. and a gentamicin sulfate solution (50 mg/ml) was added prior to inoculation into the allantoic cavity of five, 10-day-old specific pathogen free embryonated chicken eggs (100 μl/egg). Inoculated embryos were incubated at 37°C for 24-48 h.

### Haemagglutination (HA) and Avian influenza virus antigen detection

The allantoic fluid of pooled infected embryos was subjected to haemagglutination in a 25-μl volume in 96-well-haemagglutionation-plate as described [15]. HA positive allantoic fluid was screened for the AIV group and H5 antigens using the rapid chromatographic strip test (Animal Genetic Inc. Korea) according to manufacturer instructions. Negative and positive controls were included along with the tested samples.

### Viral RNA extraction and RT PCR

The extraction of viral RNA was conducted from a virus containing allantoic fluid using a spin column purification kit (Koma Biotech. Inc., Korea). The influenza subtype was first screened using H5, H7, H9, N1, N2 and N3 specific primers (Table 1). Amplification of internal genes was performed with gene-specific primers for the six viral genes: PB2, PB1, PA, HA, NP, NA, M and NS (Table 1) using a Koma one step RT PCR kit (Koma Biotech. Inc., Korea). PCR amplicons were subjected to electrophoresis in a 1.5% agarose gel. Specific bands of expected sizes were excised and purified using a QIAquick gel extraction kit (Qiagen, Germany). Purified RT-PCR products were sequenced directly in both forward and reverse directions (Macrogen, Korea). Different gene sequences were assembled by trimming primer-linker. Sequence data of the H9N2 Egyptian strain isolated in the current study are available in the GenBank database (Accession No: JQ611701 - JQ611708).

**Table 1:** Oligonucleotides used for amplification of the eight genes of the H9N2

**Locus**	**Name**	**Primer sequence**	**Length**	**Amplicon size (bp)**	**Primer position**	**Reference**
H9	H9-F	CACAATGTMAGYAARTATGC	20	554	943-962	[16]
	H9-R	TCCATGCAYTGRTYRTCACA	20		1496-1515	
H5	H5-Fb	GAGCAGAATAAAYCATTTTGAGA	15	699	392-414	FLI^a^
	H5-Rb	TGAGTGGATTCTTTGTCTGCAGC	20		1167-1189	
H7	H7-F	ATYAAYMSYAGRRCWGTRGG	20	241	937-956	[16]
	H7-R	GATCWATTGCHGAYTGRGTG	20		1177-1196	
N3	N3-F	AGATCRGGCTTTGAARTCATCAAAGT	26	247	1126-1151	[17]
	N3-R	CATTGTCTARTCCACAGAAAGTAACTATAC	30		1372-1343	
N2	N2-F	GCATGGTCCAGYTCAAGYTG	20	362	548-567	[17]
	N2-R	CCYTTCCAGTTGTCTCTGCA	20		909-890	
N1	N1-F	ATGAATCCAAATCAGAAG	18	1350	21-38	[13]
	N1-R	TGTCAATGGTGAATGGCAAC	20		1346-1365	
PB2	B2-F	TATTCATCRTCAATGATGTGGGA	23	540	1591-1613	[18]
	B2- R	GATGCTYAATGCTGGTCCATATC	23		2130-2108	
PB1	B1-F	AGCGAGGAGTATCTGTGAGA	21	601	774-793	This study
	B1-R	TTCCCTCATGATTCGGTGCA	20		1356-1375	
PA	PA-F	CCCATGTTCCTGTATGTGAG	20	456	1678-1697	This study
	PA-R	GAGGAAGGAGTTGAACCAAG	20		2114-2133	
NP	NP-Fs	TGCTTGCCTGCTTGTGTGTA	20	665	823-842	[19]
	NP-Rs	TACTCCTCTGCATTGTCTCCGA	22		1466-1487	
M	M-F	CCCTCAAAGCCGAAATCGCGCA	22	875	56-77	This study
	M-R	TGCTGTTCCTGCCGATACTCTTCCC	25		906-930	
NS	NS-F	CACTGTGTCAAGCTTTCAGG	20	798	23-42	[19]
	NS-R	TCTCTTGCTCCACTTCAAGC	20		786-805	

^a^Primer sequences for HA5 were ordered according to information provided by the Laboratory of Molecular and Biological Characterization of AIV, Friedrich Loeffler Institute (FLI), Germany.

### Genetic and phylogenetic analysis

Sequence analysis of the viral genes was conducted using Mega 4.1 as previously described [20]. Sequence alignments were constructed for the partial coding regions of each of the eight genomic segments. For comparison, the multisequence and phylogenetic analyses of the isolated H9N2 strain in the current study, the H9N2 virus strain that was isolated from Egypt on 2006 (All sequences except HA gene are available), and the recently isolated Egyptian strain from quail [21] as well as different H9N2 virus lineages circulated in the Middle East together with the ancestor Asian strains for both H9 and N2 genes were included. On the other hand, we screened the possible reassortment in the isolated H9N2 strain in comparison to different H9N2 strains. All gene sequence data were collected from the National Center for Biotechnology Information (NCBI) flu database. The neighbour-joining method with Kimura two-parameter distances was used for constructing the phylogenetic trees using the Mega 4.1 [22]. The reliability of the internal branches was assessed by the p-distance substitution model and 1000 bootstrap replications.

### Deduced amino acid sequence analysis

The multisequence alignment tool available in the flu database was used to compare the deduced amino acid sequences of the six internal genes of the Egyptian H9N2 strain with other H9N2 strains from the Middle East, and Egyptian H5N1 isolates available in the flu database as well as the H1N1 2009 pandemic strain in order to screen amino acid signature and mutation trend change. Amino acid residues that have associated with mammalian virulence were also screened.

## Results and discussion

**Figure 1:**
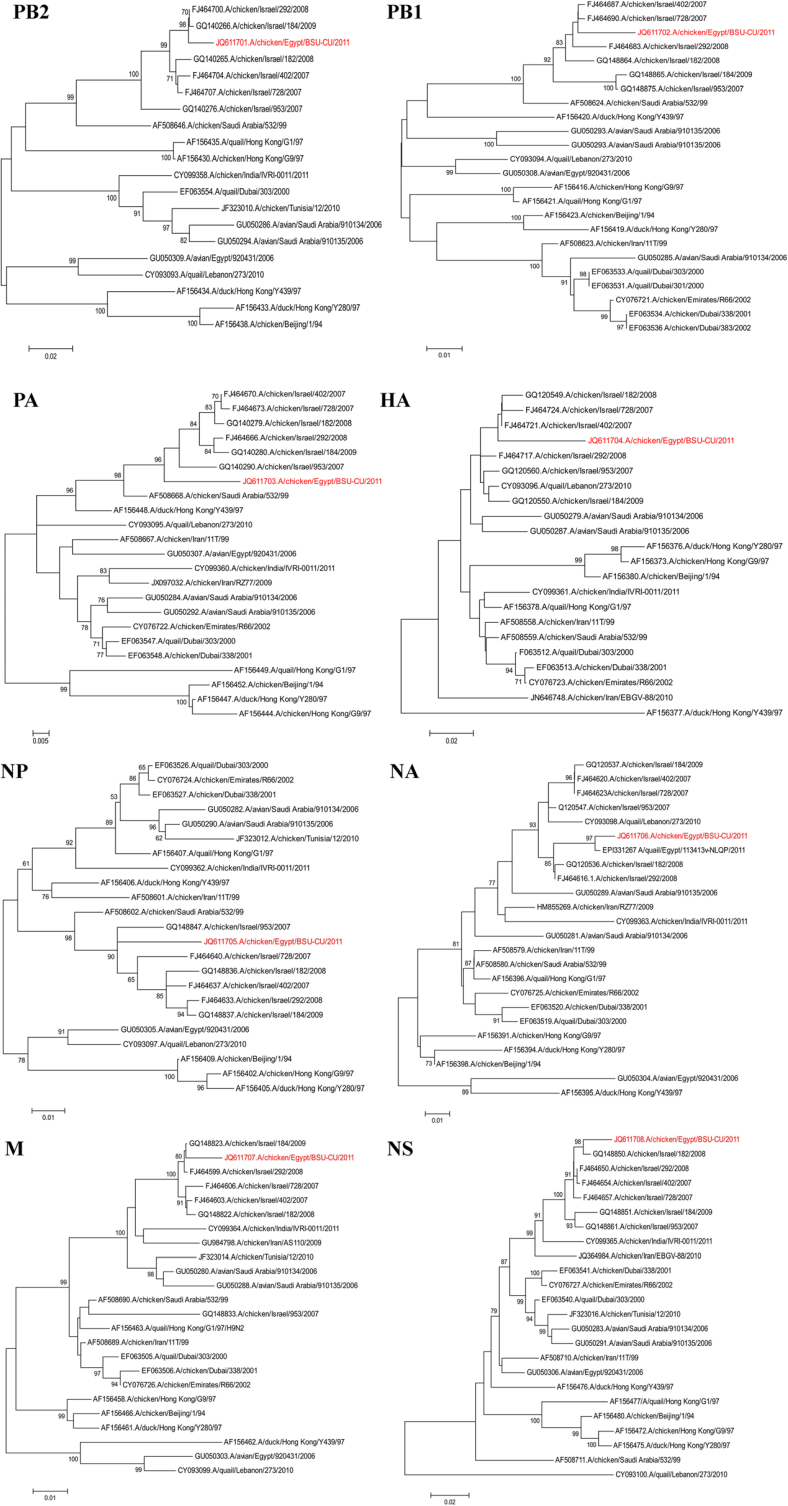
**Phylogenetic analysis of the PB1, PB2, PA, HA, NP, NA, M and NS genes of the A/chicken/Egypt/BSU-CU/2011 and selected H9N2 influenza A virux ses from the GenBank and EMBL databases.** The strain isolated in the current study is indicated in red. The selected viruses were chosen to be representative of the relevant sequences in the GenBank and EMBL databases. The robustness of individual nodes of the tree was assessed using 1000 bootstrap replicates, and bootstrap values 70% and higher are indicated at key nodes.

In the current study, we described the isolation of H9N2 from chicken flocks suffering from respiratory distress with morbidity reached 70% and mortality reached 15%. The virus was isolated from the Northern part of Egypt; the Alexandria Governorate. The suspected materials from the infected birds induced embryo death after 48 h of inoculation. The infective allantoic fluid was able to agglutinate chicken red blood cells and reacted positive with the AIV group but not H5 antigens using the rapid chromatographic strip test. It also reacted negatively upon testing with RT-PCR using H7, H5, N1, N3 specific primers (data not shown). Positive results were obtained upon testing with RT-PCR using H9 and N2 specific oligonucleotides. Blast analysis of the nucleotide sequences from the eight viral genes showed that the recently isolated Egyptian H9N2 strain, A/chicken/Egypt/BSU-CU/2011, was closely related to the other Middle East H9N2 strains. The virus shared the common ancestor A/Qa/HK/G1/97 isolate which has contributed the internal genes of the H5N1 virus circulating in Asia. The NA gene of A/chicken/Egypt/BSU-CU/2011 is closely related to the newly isolated Egyptian quail strain [21] (Figure 1), both were isolated on May 2011 in a consequent manner: 5^th^ May and 28^th^ May 2011 for chicken and quail isolates respectively. Meanwhile, it seems that the H9N2 circulated in Egypt in undetectable manner as we recorded a serological evidence of H9 spread throughout Egypt on 2009-2010 [23]. We could not align the HA of the isolated chicken strain in the current study with the Egyptian quail strain due to the difference in the amplicon sites on the target gene, however, both were found closely related to the Israeli strains (2007-2009).

The evolutionary trees of the PB2, PB1, PA, HA, NP, NA, M and NS genes revealed that the alleles corresponding to the viral segments of the A/chicken/Egypt/BSU-CU/2011 isolate were all Eurasian in origin (Figure 1). The phylogenetic analysis suggested that all the eight alleles of the A/Egypt/chicken/BSU-CU/2011 were closely related to Israeli isolates (2007-2009) with an average percent pairwise nucleotide distances less than <5%. Accordingly, they possess the same subtype of Israeli isolates based on the genome constellations of the H9N2 viruses described by Fusaro et al. [7]. On the other hand, the PB2, HA, NA, M alleles of the A/chicken/Egypt/BSU-CU/2011 are derived from the G1 lineage, while the PB1, PA, NP, NS are not related probably due to the inter-subtype reassortment (Figure 1). According to Fusaro et al. typing of H9N2 strains circulated in the Central Asia and the Middle East, the PB2, PB1, PA and NP gene segment constellation of A/chicken/Egypt/BSU-CU/2011 related to group A segment constellation while HA, NA, M and NS are related to group B segment constellation [7].

Understanding the genetic characteristics and mutation trend of the H9N2 virus isolated in Egypt provides clues to understand the ecology of H9N2. The haemagglutinin cleavage motif sequence of the A/Chicken/Egypt/BSU-CU/2011 was found to be PARSSR/GLF; thus, confirming the low pathogenic nature of the Egyptian H9N2 strain however, it was similar to the RX-RYK-R [24]. This genetic constitution suggested that the current H9N2 strain may have the potential to acquire basic amino acids in the HA connecting the peptide sequence needed to become highly pathogenic. The virus neuraminidase showed high homology to the Egyptian quail strain and differed only in a single amino acid substitution (data not shown) and both found closely related to Israeli H9N2 2008 strains (Figure 1).

The PB2 protein of the A/Chicken/Egypt/BSU-CU/2011 displayed valine (V) at position 627 (Tables 2 and 3), two avian like amino acids at positions 661 alanine (A) (data not shown) and lysine (K) (Table 3) found in the functional domain that is responsible for the interaction with other polymerase components [25].

**Table 2:** Comparison of amino acid signatures in selected genes of avian and human strains to Egyptian H9N2 and H5N1 strains

**Gene**	**Residue**	**Avian**^**a**^	**Human**^**a**^	**H1N1 Pandemic**		**Egyptian H5N1**	**A/ck/Eg/BSU-CU/11**	**Egypt. H9N2 2006**^**b**^	**Middle East H9N2**^**c**^
				**2009**	**1918**				
PB2	588	A^203^/T^6^/V^6^	I^835^/V^3^/A^2^	T	A	A^32^/T^1^	A	A	A^82^/V^10^/I^1^
	613	V^212^/A^3^	T^816^/I^16^/A^8^/V^1^	V	V	V^33^	V	V	V^92^/I^1^
	627	E^196^/K^19^	K^838^/R^2^/E^1^	E	K	K^32^/E1	V	E	V^60^/E^31^/A^2^
	674	A^204^/S^6^/T^2^/G^2^/E^1^	T^836^/A^2^/I^2^/P^1^	A	A	A^33^	A	A	A^88^/T^5^
PB1	327	R^147^/K^3^	K^766^/R^66^	R	R	R^35^	R	R	R^81^
	336	V^142^/I^8^	I^773^/V^59^	I	V	V^35^	V	V	V^81^
NP	283	L^372^/P^1^	L^7^/P^643^	L	P	L^33^	L	L	L^78^
	293	R^371^/K^2^	R^28^/K^622^	R	R	R^32^/K^1^	R	R	R^78^
	305	R^369^/K^4^	K^636^/R^14^	F	L	R^33^	R	R	R^78^
	313	F^371^/I^1^/L^1^	Y^642^/F^8^	V	Y	F^33^	F	F	F^78^
	357	Q^368^/K^4^/T^1^	K^44^/R^8^/Q^1^	K	K	Q^33^	Q	Q	Q^76^/K^2^
	372	E^357^/D^15^/K^1^	D^630^/E^23^	E	E	E^33^	E	E	E^72^/D^6^
	422	R^373^	K^630^/R^23^	R	R	R^33^	R	R	R^78^
	442	T^372^/A^1^	A^629^/T^23^/R^1^	T	T	T^33^	T	T	T^78^
	455	D^373^	E^630^/D^22^/T^1^	D	D	D^32^/E^1^	D	D	D^78^
M1	115	V^856^/I^2^/L^1^/G^1^	I^981^/V^9^	V	V	V^35^	V	V	V^94^
	121	T^840^/A^19^/P^1^	A^988^/T^2^	T	A	T^35^	T	T	T^94^
	137	T^859^/A^1^/P^1^	A^974^/T^12^	T	T	T^35^	T	T	T^94^
M2	11	T^434^/I^11^/S^2^	I^911^/T^44^	T	T	T^35^	T	T	T^90^/S^1^
	20	S^471^/N^13^	N^926^/S^29^	S	N	S^35^	S	S	S^83^N^6^G^1^
	57	Y^481^/C^1^/H^1^	H^913^/Y^33^/R^2^/Q^1^	Y	Y	Y^35^	Y	Y	Y^87^H^2^
NS2	70	S^453^/G^21^/D^1^	G^903^/S^2^	G	S	S^52^/G^7^	S	S	S^83^G^13^

^a^Avian and human amino acid signatures in different viral genes of influenza A viruses as previously determined [26].

^b^A/avian/Egypt/920431/2006(H9N2)) is GenBank available data. The strain isolated from live bird market in Egypt (unpublished report).

^c^All available H9N2 sequences from the Middle East. The available H9N2 sequences include strains from Emirates, Iran, Israel, Jordan, Lebanon, Saudi Arabia and Tunisia found in the Flu database.

Numerical superscripts refer to the number of strains that possess those residues.

**Table 3:** Amino acid site residues associated with virulence in mammals in comparison with Egyptian isolates

**Gene**	**Site**	**Virulent**	**Avirulent**	**A/ck/Eg/BSU-CU/11**	**Egypt H9N2 2006**^**b**^	**Middle East H9N2**	**Egyptian H5N1 isolates**	**Reference**
PB2	627	K	E	V	E	V^60^/E^31^/A^2^	K	[27,28]
	701	N	D	D	D	D	D	[29]
PB1	317	I	M/V	I	M	M	M/V	[27,28]
M2	64	S/A/F	P	S	S	S	S	[30]
	69	P	L	P	P	P	P	[30]
NS1	42	S	A/P	S	S	S	S	[31]
	92	E	D	D	D	D	D	[28]
	97/92	E	D	E	E	E	E	[32]
	127	N	T/D/R/V/A	T	N	N^57^/T^42^	T/I	[33]
	189	N	D/G	D	D	D^98^/N^1^	D	[34]
	195	T/Y	S	S	S	S	S	[35]
NS2	31	I	M	I	I	I	M	[34]
	56	Y	H/L	H	H	H^98^/Y^1^	H	[34]

^a^Virulent and non virulent amino acid residues refer to the ability of the virus to replicate in mammals as determined by Lycett et al. [30].

^b^A/avian/Egypt/920431/2006(H9N2)) is GenBank available data. The strain isolated from live bird market in Egypt (unpublished report).

^c^All available H9N2 sequences from the Middle East. The available H9N2 sequences include strains from Emirates, Iran, Israel, Jordan, Lebanon, Saudi Arabia and Tunisia found in the Flu database.

Numerical superscripts refer to the number of strains that possess those residues.

Amino acid substitutions at one of the M2 residues; 26, 27, 30, 31, 34, or 38 could lead to amantadine resistance [36,37]. None of such amino acid substitutions were found in the Egyptian strain (data not shown). De Filette et al., delineated M2 specific sequences specific for human, swine and avian species [38]. In comparison to the current Egyptian isolate, it was noted that it possessed three human specific residues; E14, G16 and R18 [38,39]. Interestingly, asparagine (N13) to a serine (S) substitution was detected in the M2 of both the Egyptian strain (Table 4) and the pandemic H1N1 strains [40].

**Table 4:** Comparison of the deduced amino acid sequence of M2 of the A/chicken/Egypt/BSU-CU/2011 with other influenza A viruses

**M2 Residue**	**Avian**^**a**^	**Human**^**a**^	**2009 H1N1 Pandemic strain**	**Egyptian H5N1**	**A/ck/Eg/BSU-CU/11**	**Egypt H9N2 2006**^**b**^	**Middle East H9N2**^**c**^
10	P	P	P	P	L	P	L^87^/P^3^
11	T	I	T	T	T	T	T^89^/S^1^
13	N	N	S	N	S	N	N^90^
14	G	E	E	E	E	G	G^57^/E^30^/A^3^
16	E	G	E	E	G	E	G^84^/E^3^/D^3^
18	K	R	R	R	R	K	R^88^/K^3^
20	S	N	S	S	S	S	S^83^/N^6^/G^1^

^a^Avian and human amino acid residues allocated by de Filette et al. [38].

^b^A/avian/Egypt/920431/2006(H9N2)) is GenBank available data. The strain isolated from live bird market in Egypt (unpublished report).

^c^All available H9N2 sequences from the Middle East. The available H9N2 sequences include strains from Emirates, Iran, Israel, Jordan, Lebanon, Saudi Arabia and Tunisia found in the Flu database.

Numerical superscripts refer to the number of strains that possess those residues.

Many studies have reported that the NS1 protein suppressed the host antiviral defenses at multiple levels and were correlated to virulence [31,41]. Sequence analysis of the non structural protein gene (NS1) of the Egyptian strain showed that it did not have the five amino acid (TIASV) deletions and contained the "KSEV" PDZ ligand (PL) C-terminal motif. It is apt to mention that increasing the length of the 2009 H1N1 NS1 protein to 230 aa did not increase the virus replication in human and pig cells [42]; however, NS1 with 5 amino acid deletion residues showed increased virulence in both mouse and poultry [32]. Another study reported that NS1 with truncations were found to stimulate more interferon than viruses with full- length NS1 proteins and were consequently more attenuated in mice [43]. The Egyptian strain: A/chicken/Egypt/BSU-CU/2011, possessed PDZ ligand C-terminal KSEV domain (data not shown). The C-terminal domain of NS1 functions as a species-specific virulence domain. Most of the avian influenza viruses possess an NS1 protein with a PDZ ligand C-terminal ESEV domain whereas human viruses have a conserved RSKV domain. NS1 proteins with C-terminal ESEV, KSEV, and EPEV domains were shown to bind to PDZ domains containing cellular proteins [44]. Viruses that possess RSKV motif, which lacks a PDZ- binding domain, replicated more efficiently than those harbouring ESEV in human and duck cells [45]. This finding suggested that the ability of NS1 to interact with PDZ-contained proteins did not contribute to the virulence [46]. Nevertheless, insertion of four C-terminal aa, either ESEV, KSEV, or EPEV, into avirulent viruses resulted in an increase in virus virulence [46]. The RNA-binding domain of the Egyptian H9N2 displayed amino acid residues proline (P31), asparagine (D34), arginine (R35), (R38), lysine (K41), glycine (G45), arginine (R46) and threonine (T49). R38 and K41, which were found to be critical for RNA binding as well amino acid residues P31, D34, R35, G45, R46, T49 and D55, also mediated NS1-dsRNA interaction [47]. Amino acid substitutions: alanine (A) or proline(P) to serine (S) at position 42 and asparagine (D) to glutamic acid (E) at positions 97 were found in the Egyptian H9N2 strain. These mutations were among those responsible for the virulence of H5N1 in mammalian species and cytokine resistance [48]. In addition, amino acid substitutions: leucine (L) to phenylalanine (F) at position 103 and isoleucine (I) to methionine (M) at position 106 were found to be adaptive genetic determinants for growth, and virulence in both mammals and avian NS1 genes [49]; interestingly, the Egyptian strain harboured both of these amino substitutions. The Egyptian strain also harboured glycine (G184) in addition to its contribution to cleavage and polyadenylation specificity factor binding; G184 strongly affected the viral virulence by an unknown mechanism [50]. Among the Middle East H9N2 available strains, the A/chicken/Egypt/BSU-CU/2011 showed unique amino acid changes at leucine (L98) and threonine (T180) of the NS1 protein.

In addition we have compared the amino acid residues associated with H5N1 virulence in mammals [27-35] to its corresponding residues in the A/chicken/Egypt/BSU-CU/2011 as well as the Middle Eastern H9N2 strains. The A/chicken/Egypt/BSU-CU/2011 possessed virulent amino acid substitutions in: PB1, M317 to I; NS1, A41 to S, E97 to D; NS2, M31 to I; M2, P64 to S and L69 to P. All the detected virulent residues are also found in the H9N2 strains from the Middle East and Egyptian H5N1 strains. Interestingly, H9N2 in the current study possessed the virulent residue I317 in the PB1 protein (Table 3); this virulent residue has never been seen in the Egyptian H5N1 isolates.

Chen et al., delineated avian and human amino acid signatures in different viral genes of influenza A viruses [26]. In the current study we have compared some of such amino acid signatures to the A/chicken/Egypt/BSU-CU/2011, H9N2 strains in the Middle East, H1N1 strains from 1918 and 2009 pandemics as well as the Egyptian H5N1 strains that were available in the GenBank for the five internal genes (PB2, PB1, NP, M and NS). We have found that the amino acid signatures in the A/chicken/Egypt/BSU-CU/2011 and H9N2 viruses circulated in the Middle East and most of the Egyptian H5N1 have possessed avian like amino acid signatures (Table 2). G9 and G1 like viruses as well as H9N2 viruses that were isolated in Hong Kong in 2003 were found to have the ability to replicate systemically and are lethal for mice [3]. Most of the viruses that contain G1-like genes were able to replicate in the mouse lungs without prior adaptation [51]. Meanwhile, Li et al., determined amino acid substitutions associated with the virulence of H9N2 viruses to mice [51]: PA F672 to L; NP, K398 to Q; M1, A195 to S; NS1, R25 to Q, A225 to T, and NS2, T14 to M substitutions were detected in the A/chicken/Egypt/BSU-CU/2011 and the Middle East H9N2 strains (Table 5).

**Table 5:** Amino acid residues of the H9N2 viruses that correlate with replication in mammals in comparison to A/chicken/Egypt/BSU-CU/2011 residues

**Gene**	**Residue No.**	**Non Virulent**^**a**^	**Virulent**^**a**^	**A/ck/Eg/BSU-CU/11**	**Egypt H9N2 2006**^**b**^	**Middle East H9N2**^**c**^
PA	672	F	L	L	L	L
NP	398	K	Q	Q	Q	Q
M1	195	A	S	S	S	S
M2	14	E	G	E	G	E^30^A^3^G^59^
NS1	25	R	Q	Q	Q	Q
	225	A	T	T	T	A^1L^R^1r^T^69^
NS2	14	T	M/I/K	M	M	K^4^M^31^L^5^V^2^Q^2^

^a^Virulent and non virulent amino acid residues refer to the ability of the virus to replicate in mice as determined by Li et al. [51].

^b^A/avian/Egypt/920431/2006(H9N2)) is GenBank available data. The strain isolated from live bird market in Egypt (unpublished report).

^c^All available H9N2 sequences from the Middle East. The available H9N2 sequences include strains from Emirates, Iran, Israel, Jordan, Lebanon, Saudi Arabia and Tunisia found in the Flu database.

Numerical superscripts refer to the number of strains that possess those residues.

## Conclusion

The genetic characteristics and mutation trend of the H9N2 virus isolated from chicken in Egypt was described. The increased mortality that associated with the disease described here, might dente additional factors that affected the case severity and allowed the LPAI-H9N2 virus to cause severe signs like. The study provided evidences to further understand the mutation trend relevant to inter-species transmission of H9N2 influenza A viruses. Genetic characterization of influenza viruses circulating in Egypt is critically important to provide insight into the key features for emergence of H9N2 among poultry and as a potential pandemic virus.

## Competing interests

The authors declare that they have no competing interests.

## Authors’ contributions

ASA carried out all the experiments including designing the experiments, acquisition of data, analysis and interpretation of data, and they drafted the manuscript. MAA and MFE have helped in the acquisition of samples, data analysis and revising the manuscript. All authors have read and approved the final manuscript.
